# Reply to Budowle, Ge, Chakraborty and Gill-King: use of prior odds for missing persons identifications

**DOI:** 10.1186/2041-2223-3-2

**Published:** 2012-01-31

**Authors:** Alex Biedermann, Franco Taroni, Pierre Margot

**Affiliations:** 1 Faculty of Law and Criminal Justice, School of Criminal Justice, Institute of Forensic Science, University of Lausanne, le Batochime, 1015 Lausanne-Dorigny, Switzerland

**Keywords:** prior probabilities, Bayesian inference, missing persons identifications

## Abstract

Prior probabilities represent a core element of the Bayesian probabilistic approach to relatedness testing. This letter opinions on the commentary *Use of prior odds for missing persons identifications *by Budowle et al., published recently in this journal. Contrary to Budowle et al., we argue that the concept of prior probabilities (i) is not endowed with the notion of objectivity, (ii) is not a case for computation, and (iii) does not require new guidelines edited by the forensic DNA community--as long as probability is properly considered as an expression of personal belief.

## 

'What is the probability that these (unidentified) human remains are those of this missing person?' This question represents a typical example of Bayesian inference: an initial state of belief, expressed in terms of probabilities (or, alternatively odds) based on preliminary circumstantial information, can be revised through scientific data--in particular results of DNA analyses. As noted by Budowle et al. [1] in their commentary *Use of prior odds for missing persons identifications*, a reasoner's opinion after the consideration of genetic data is a function of the initial state of belief, so that there is some interest in inquiring about its foundations. Budowle et al. [1] further note that, besides considering the total number of missing persons, the field has particularly been silent about how to set initial probabilities. Their conclusion thus is that '[t]he forensic DNA community needs to develop guidelines for objectively computing prior odds' [[1], p. 1]. It is on this particular conclusion that we wish to comment here. In particular, we seek to argue that by considering a reasoner's belief state through the personalist interpretation of probability, the topic of prior odds (1) does not relate to objectivity, (2) is not a case for computation, and (3) does not require new guidelines.

(1) The notion of objectivity is as widely used in scientific communications as it is undefined and, after all, illusionary [2]. In the particular context of forensic DNA analyses, Evett and Weir concisely expressed this as follows:

'(. . .) we do not accept that DNA statistics are objective in the sense of being independent of human judgment. In spite of the often elegant mathematical arguments we have presented, we stress that the final statistical values depend wholly on the initial assumptions. The validity of these assumptions in any given case are a matter for expert opinion, so that we claim "objective science" can exist only within the framework of subjective judgment.' [[3], p. 217]

Perceived objectivity thus only exists within a framework of intersubjectively accepted assumptions so that, at best, one may say that '[o]bjectivity is merely subjectivity when nearly everyone agrees' [[4], p. 87]. Here, the term 'subjective' means 'personal' because it refers to the state of belief of an individual reasoner--there is no suggestion of 'bias' or 'arbitrary'.

(2) According to the personalist (or belief-type) interpretation, probability is not endowed with any sort of objective significance that could be seen as a property of the external world and that exists independently of the reasoning individual. The idea of 'computing', however, precisely suggests the independent existence of a rule or procedure that could dictate to reasoners, based on some aspects of the problem at hand, what ought to be their initial probabilities. This is exemplified by the recommendation that, in a case with *v *victims, along with an assumption of equiprobability, a reasoner's prior probability ought to be 1/*v*. Although the reasoning individual is free to choose such a technical and artificial constraint on a voluntary basis, this amounts to a subjective choice and does in no way represent a necessary requirement that stems from the theory of probability [5]. The laws of probability only specify the range of values that probabilities can take, and how probabilities are to be combined [6]. More generally, a computational approach to prior probability assignment is misconceived because it imposes a technocratic view on a topic that is actually a case of opinion. Taking a broad view can help to see this in context, as argued by Lindley when he wrote that '(. . .) the model is your description of the uncertainty present in your perception of the situation, the uncertainty expressed in terms of probability. Thus, the prior is (. . .) part of the model (. . .). Once the model is settled in this complete form, technique is able to take over; but not until then. Technique cannot produce an opinion (. . .)' [[7], p. 184].

(3) If, following Budowle et al., '[t]he prior probability should reflect reasonable beliefs about an event before receiving new information' [[1], p. 3]--a viewpoint that we unreservedly support--then this is a case for the personalistic approach to probability. Its successful implementation relies on an awareness among reasoners of their very own personal opinions, as well as their nature. A way ahead thus is a genuine understanding of the concept of probability [8] which, if necessary, appropriate education may favor [9]. A development of guidelines, in turn, could amount to or suggest a rigid application of 'recipes' that deprive the decision maker's mind of any ductility.

Should the forensic DNA community elaborate on *new *guidelines? While we concede that guidelines can have the merit of expressing 'intersubjectively' agreed suggestions of relevant factors and precautions that ought to be taken into account in prior probability assignment, along with a possibly educational component, it remains questionable whether scientists should interfere with a topic of which practicing legal decision makers are already well aware and that, above all, they are in a better position to appreciate (e.g., [10]).

## Competing interests

The authors declare that they have no competing interests.

## Authors' contributions

All authors contributed equally to this manuscript and read and approved the final manuscript.
